# Additive Effects of Monetary Loss and Positive Emotion in the Human Brain

**DOI:** 10.1523/ENEURO.0374-23.2024

**Published:** 2024-04-12

**Authors:** Sagarika Jaiswal, Lakshman N.C. Chakravarthula, Srikanth Padmala

**Affiliations:** Centre for Neuroscience, Indian Institute of Science, Bangalore, Karnataka 560012, India

**Keywords:** additive integration, conflicting outcomes, experienced value, monetary losses, positive emotion, ventromedial prefrontal cortex

## Abstract

In many real-life scenarios, our decisions could lead to multiple outcomes that conflict with value. Hence, an appropriate neural representation of the net experienced value of conflicting outcomes, which play a crucial role in guiding future decisions, is critical for adaptive behavior. As some recent functional neuroimaging work has primarily focused on the concurrent processing of monetary gains and aversive information, very little is known regarding the integration of conflicting value signals involving monetary losses and appetitive information in the human brain. To address this critical gap, we conducted a functional MRI study involving healthy human male participants to examine the nature of integrating positive emotion and monetary losses. We employed a novel experimental design where the valence (positive or neutral) of an emotional stimulus indicated the type of outcome (loss or no loss) in a choice task. Specifically, we probed two plausible integration patterns while processing conflicting value signals involving positive emotion and monetary losses: interactive versus additive. We found overlapping main effects of positive (vs neutral) emotion and loss (vs no loss) in multiple brain regions, including the ventromedial prefrontal cortex, striatum, and amygdala, notably with a lack of evidence for interaction. Thus, our findings revealed the additive integration pattern of monetary loss and positive emotion outcomes, suggesting that the experienced value of the monetary loss was not modulated by the valence of the image signaling those outcomes. These findings contribute to our limited understanding of the nature of integrating conflicting outcomes in the healthy human brain with potential clinical relevance.

## Significance Statement

In everyday life, our decisions could lead to outcomes that involve both positive and negative values—for instance, getting well paid when working under hostile conditions. Hence, an appropriate neural representation of the net value of such conflicting outcomes plays an important role in guiding future decisions. However, our current understanding of how the human brain integrates value signals from conflicting outcomes is rudimentary. Using functional MRI, we investigated the nature of integrating positive emotion and monetary losses. We found that both positive emotion and monetary losses recruited the same brain regions but their effects were largely independent of each other. These findings contribute to a small but growing literature on the integration of conflicting outcomes in the human brain.

## Introduction

In many real-life scenarios, our decisions could lead to multiple outcomes that conflict with value. For instance, getting well paid when working under hostile conditions. Hence, an appropriate neural representation of the net experienced value of conflicting outcomes, which plays a crucial role in guiding future decisions, is critical for adaptive behavior (Marien et al., 2013). Past studies that examined the neural mechanisms underlying the integration of value signals have mainly focused on the net anticipatory value of conflicting outcomes (Talmi et al., 2009; Choi et al., 2014; Yee et al., 2021). However, the anticipatory and consummatory phases of value processing are distinct psychological processes (Berridge et al., 2009) and engage partially dissociable neural substrates (Bartra et al., 2013; Oldham et al., 2018). Therefore, it is crucial to separately examine the neural substrates underlying the net experienced value of conflicting outcomes.

A recent fMRI study has investigated the concurrent processing of monetary gains and aversive stimulation outcomes (Kim and Anderson, 2020). The authors reported that monetary gains recruited the ventral striatum and medial prefrontal cortex, while the anterior cingulate cortex and anterior insula were sensitive to aversive stimulation. Contrary to the author's predictions, monetary gain activity was not attenuated in the presence of concurrent aversive stimulation or vice versa, revealing little or no evidence for the integration of these two conflicting outcomes. Despite these recent null findings, several previous studies (Smith et al., 2010; Wake and Izuma, 2017; Oren et al., 2022) have reported that outcome-related fMRI activity in the ventromedial prefrontal cortex (vmPFC) and striatum is sensitive to the entire range of value dimension (i.e., negative to positive) irrespective of the outcome modality (e.g., monetary, emotional, food), suggesting there is potential for integrating conflicting value signals from diverse outcome categories.

More importantly, as the recent work primarily focused on the concurrent processing of monetary gains and aversive information, very little is known regarding the integration of conflicting value signals involving monetary losses and appetitive information (Bandyopadhyay et al., 2019). This complementary scenario is important to investigate as the subjective experience of losses could differ from the gains (Sokol-Hessner and Rutledge, 2019). Furthermore, the underlying neural substrates might be distinct (Liu et al., 2011; Chen et al., 2022) and highly context dependent (Seymour et al., 2015). Hence, examining how concurrent appetitive information modulates the evaluation of loss outcomes would help us gain a broader understanding of the neural processing of conflicting outcomes.

**Figure 1.**
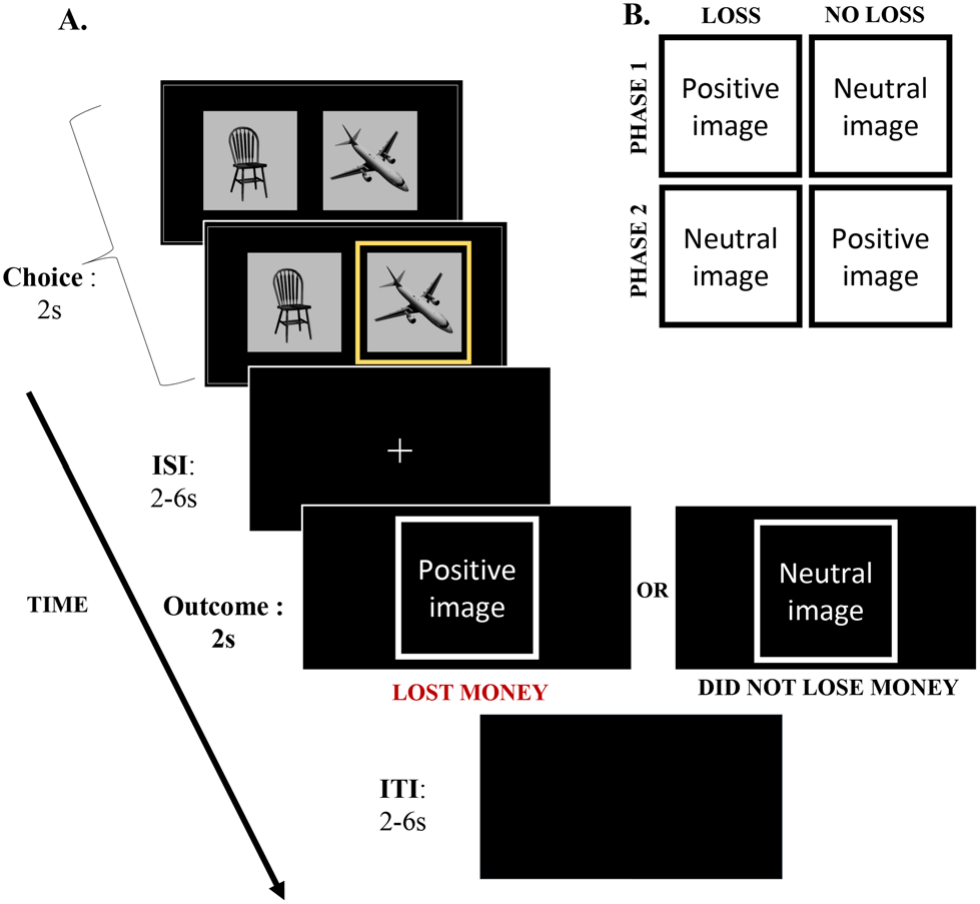
Experimental design. ***A***, On each trial, during the initial choice stage, participants viewed images of chairs and planes displayed side-by-side on the screen for 2 s and were asked to select one of those images. A yellow-colored square highlighted the selected choice. After a jittered ISI, an emotional image (positive or neutral) was shown as feedback during the subsequent outcome stage (2 s). The valence of the emotional image signaled monetary loss or no-loss outcome. In the example trial shown, if participants saw a positive image, it indicated that they lost money on that trial, while neutral indicated no loss. This emotion–loss mapping was reversed in the other phase. Each trial ended with a blank jittered ITI period. ***B***, In one phase (Phase 1), positive stimulus indicated a loss outcome, and neutral stimulus indicated a no-loss outcome. This valence–outcome mapping was reversed in the second phase (Phase 2). Each participant completed both phases of the experiment, and the order of phases was counterbalanced across participants. ISI, interstimulus interval; ITI, intertrial interval.

To address this critical gap, we conducted an fMRI study to investigate the integration of positive emotion and monetary losses using a novel experimental design where the valence of an emotional stimulus indicated the type of monetary outcome in a choice task (Fig. 1). During the initial choice stage, participants chose between two distinct categories of images, to minimize monetary losses. A positive or neutral valenced image was shown during the subsequent outcome stage, indicating whether their choice led to loss or no loss. Specifically, in one experimental phase, a positive image indicated monetary loss, whereas a neutral image indicated no loss. This instructed emotion–loss mapping was reversed in the other phase, resulting in a 2 *Emotion* (positive, neutral) × 2 *Loss* (loss, no-loss) within-subject factorial design. Our design mandated the processing of the valence (positive vs neutral) of an emotional image to determine the type of outcome (loss or no loss), thus promoting the integrated processing of conflicting monetary and emotional outcomes.

We were particularly interested in examining the nature of integrating positive emotion and loss outcomes in the vmPFC, striatum, and amygdala, which have been frequently implicated in processing monetary and emotional outcomes (Bartra et al., 2013; Sescousse et al., 2013). Specifically, we probed two plausible integration patterns while processing conflicting value signals involving positive emotion and monetary losses (Talmi and Pine, 2012). The interactive integration pattern indicates that positive emotion modulates the experienced value of monetary loss. Specifically, we expected that the negative value of loss (vs no-loss) outcomes would be attenuated when signaled by a positive compared with a neutral image. Alternatively, the additive integration pattern indicates that the effects of monetary losses and positive emotion are independent, implying that the experienced value of loss (vs no-loss) outcomes would be the same regardless of the valence of the image signaling those outcomes. Although there is evidence supporting both types of integration patterns during the anticipatory phase (Talmi et al., 2009; Yee et al., 2021), the nature of integrating conflicting value signals at the consummatory phase, specifically involving monetary losses and positive emotion, remains to be investigated.

## Materials and Methods

### Participants

Thirty-seven male participants were recruited for this fMRI study through poster and email adverts. The criteria to participate included an age range of 18–35 years, being right-handed, having a normal or corrected-to-normal vision, having no color blindness, having no history of neuropsychiatric disorders or any clinically diagnosed cognitive or motor deficits, no history of substance abuse, and no MRI contraindications. All participants gave their written informed consent and were screened for MR safety before the start of the scanning session. Participants were given monetary compensation of 300 Indian rupees (base pay) for their participation and reimbursed for their travel as required. All experimental procedures were approved by the Institutional Human Ethics Committee of the Indian Institute of Science and the Central Ethics Committee of the HCG (HealthCare Global Enterprises) Hospital.

Data from two participants were excluded as one participant completed only half of the task session and the other because of excessive head motion (>8 mm). So, we included the data from the remaining 35 participants (age, 22.90 ± 4.06 years) in our final analyses. We recruited only male participants in this study as we employed erotic images to manipulate positive emotion (see below, Stimulus material), and several earlier studies in the literature had reported gender differences in response to visual sexual stimuli (Bradley et al., 2001; Rupp and Wallen, 2008). Furthermore, in a pilot study we conducted with 12 female volunteers (age, 20.08 ± 2.78 years), subjective valence ratings on a scale of 1 (unpleasant)–9 (pleasant) of erotic and neutral images were similar (positive, 6.11 ± 0.65; neutral, 5.12 ± 0.59), indicating that female participants did not perceive erotic images as pleasant compared with neutral ones.

### Task procedures

During the fMRI session, the stimuli were projected on a screen placed near the foot end of the patient table, and the participants viewed the screen while lying supine on the patient table via a mirror attached to the head coil. Participants wore noise-attenuating earplugs and were asked to keep their head still inside the scanner. Participants provided responses using the ResponseGrip acquisition device (NordicNeuroLab). The stimulus presentation and response collection scripts were written in MATLAB (version R2019b) using functions from the Psychophysics Toolbox (Brainard, 1997).

The fMRI session was divided into three tasks: (1) the loss localizer task, (2) the main experimental task involving emotion and loss manipulations, and (3) the object localizer task. In the current study, given our primary research question about the integration of positive emotion and loss outcomes, we focused only on the main experimental task. The details of the loss localizer task (which did not involve any emotion manipulation) and object localizer task (which did not involve any loss or emotion manipulations) will be discussed elsewhere as it is not relevant to the aims of the current study.

Each participant was endowed with 400 Indian rupees at the start of the experiment, from which the money they lost during the loss localizer task and the main experimental task was deducted (see below, Main loss–emotion experimental task). Participants were explicitly told that the remaining balance would be paid as a bonus amount to them at the end of the experiment.

### Main loss–emotion experimental task

We employed a fast event-related fMRI design wherein on each trial of a simple two-choice task, participants chose between images (4 × 4° of subtended visual angle) of a chair and a plane displayed side-by-side for 2 s (choice stage; Fig. 1*A*). Participants were instructed that based on their choice between the two images, they could potentially lose (2 Indian rupees per trial) or avoid losing any money. They were asked to actively make choices to minimize their monetary losses. Participants had to make the selection while the images were displayed on the screen using either the left or right thumb buttons of MRI-compatible response grips, and the chosen image was highlighted in yellow; for example, if they wanted to choose the image that appeared on the left side of the screen, they had to press the left button, and if they wanted to choose the image that appeared on the right side of the screen, they had to press the right button. The chair and plane images were presented with equal probability on the left and right sides of the center of the screen. The position of these images on every trial was predetermined using a pseudorandomized order (same for all participants) with the constraint that the same position was not repeated more than twice in a sequence.

After the choice period, a white central fixation cross appeared on the screen during the 2–6 s jittered interstimulus interval (ISI), which was followed by the outcome stage (Fig. 1*A*). During the outcome stage, a positive or neutral emotional image was presented for 2 s as feedback. The valence of the feedback image signaled participants whether they lost money or did not lose on that particular trial. In case participants did not make a selection during the initial choice period, “No Response” was shown in red as text feedback (instead of an image), and participants were explicitly told that they would lose 2 Indian rupees for each missed response. Finally, each trial ended with a 2–6 s jittered intertrial interval (ITI). The employment of jittered ISI and ITI periods allowed us to separately estimate the responses during the choice and outcome stages of the task (Serences, 2004).

The main experimental task consisted of two phases (Fig. 1*B*): during the outcome stage of one phase (Phase 1), positively valent images signaled monetary loss, and neutral images signaled no loss. The valence–outcome mapping was reversed in the other phase (Phase 2), and the order of phases was counterbalanced across participants. Before the start of each phase, participants were provided instructions explicitly indicating the valence–outcome mapping. The experimenter verbally confirmed with the participants that they clearly understood the instructed mapping for each phase. Furthermore, participants underwent a short training run (24 trials) before each phase. During the outcome stage in the training runs, participants were provided additional text feedback (1 s) after the feedback image (e.g., “POSITIVE image: LOST MONEY” in red and “NEUTRAL image: DID NOT LOSE MONEY” in white) to reinforce the instructed emotion–loss association for the phase. Participants were explicitly informed that no money would be deducted during the training runs.

Unbeknownst to the participants, the outcome on every trial was predetermined using a pseudorandomized order (same for all participants) with the constraint that the same outcome (loss or no loss) was not repeated more than thrice in a sequence. This was done to ensure an equal number of trials for the different outcome conditions of interest. Despite employing predetermined outcomes, we included an initial choice component in our paradigm [see Delgado et al. (2000) and Tricomi et al. (2004) for a similar strategy] because passive delivery of outcomes (i.e., independently of any choice or action) might not activate some regions of our interest specifically the subcortical amygdala and striatum (Elliott et al., 2003; Tricomi et al., 2004; Zink et al., 2004).

Each phase of the main experimental task was subdivided into two runs of 36 trials each, with an equal number (18 trials each) of predetermined loss and no-loss outcome trials in every run. Overall, across the two phases, a total of 144 trials were employed, yielding 36 trials for each of the four outcome conditions (barring any no-response trials) in a 2 *Emotion* (positive, neutral) × 2 *Loss* (loss, no-loss) within-subject factorial design. At the end of each run, the amount of money lost in all the runs until that point of time, along with the balance remaining in the endowed amount was shown. Since the number of loss outcome trials was predetermined in each run, a random number between 0 and 3 was subtracted to artificially introduce variability in the amount of money lost across runs. Over the entire experiment, participants retained a balance of 240 ± 6.7 (mean ± SD) Indian rupees in their endowed amount, which was paid as a bonus after the experiment (in addition to the base pay for participating in the study).

Finally, at the end of each phase, participants were asked to rate their subjective experience (on a self-paced response window) on a scale of 1–9 (1, unpleasant; 9, pleasant) for the two different valence–outcome mappings they experienced during that particular phase. The two rating questions after one phase (Phase 1) were “Please rate how you felt when the LOSS information was signaled by a POSITIVE image” and “Please rate how you felt when the NO LOSS information was signaled by a NEUTRAL image” and after the other phase (Phase 2) were “Please rate how you felt when the LOSS information was signaled by a NEUTRAL image” and “Please rate how you felt when the NO LOSS information was signaled by a POSITIVE image.”

### Stimulus material

We employed 144 images each of chairs and planes during the choice stage of the main experimental task, and none of the images were repeated. These images were collected from the Internet and converted to gray scale in MATLAB. Furthermore, these grayscaled images were normalized in luminance (to the mean of the stimulus set) using the SHINE toolbox in MATLAB (Willenbockel et al., 2010). During the outcome stage of the main experimental task phases, we employed 72 positive and 72 neutral colored emotional scene stimuli. None of these scene stimuli were repeated to prevent potential emotional habituation and loss history effects. These scene stimuli were collected from different standard databases such as the International Affective Picture System (Bradley et al., 2001; Rupp and Wallen, 2008), the Nencki Affective Picture System (Marchewka et al., 2014), the Open Affective Standardized Image Set (Chen et al., 2020), and a few were taken from the Internet. Positive stimuli depicted couples in erotic and romantic poses, and neutral ones had people involved in regular activities and some neutral scenes.

In an initial pilot study involving 11 male participants of similar age (21.82 ± 2.68 years) from the same community, we collected subjective ratings of valence and arousal of 360 stimuli (120 each from positive, negative, and neutral valence categories presented in a pseudorandom order). Participants provided ratings of their emotional experience on a valence–arousal affect grid rating scale (Russell et al., 1989), with the valence dimension on the horizontal axis ranging from 1 (unpleasant) to 9 (pleasant) and the arousal dimension on the vertical axis ranging from 1 (low arousal) to 9 (high arousal). For the current study, out of the 120 images from each valence category, we selected 72 positive stimuli with average valence ratings of >6.9 and average arousal ratings of >4.5 and 72 neutral stimuli with average valence ratings between 4.4 and 5.7 and average arousal ratings of <2.8. Then, we subdivided these 72 stimuli from positive and neutral categories into two subsets of 36 stimuli each in such a way that valence and arousal ratings differed between categories in each set but were comparable across sets (Table 1). These two nonoverlapping stimulus sets were used in two phases of the main experimental task. The assignment of a particular stimulus set to one of these phases was counterbalanced across participants. Finally, a separate set of images were used during the choice and outcome stages of the training run before each phase.

**Table 1.** Mean self-reported valence and arousal ratings (with standard deviation in parentheses) of the two sets of 36 scene stimuli each that were employed in this study

Valence ratings							
Descriptive values					Two-way ANOVA results		
	Set1		Set2		Effect	*F* value	*p* value
*Emotion*	Mean	SD	Mean	SD	Main effect: emotion	41.391	<0.001
Positive	7.19	0.22	7.16	0.19	Main effect: set	1.862	0.202
Neutral	5.22	0.29	5.16	0.25	Interaction effect: emotion*set	0.094	0.766
Arousal ratings							
Descriptive values					Two-way ANOVA results		
	Set1		Set2		Effect	*F* value	*p* value
Emotion	Mean	SD	Mean	SD	Main effect: emotion	134.683	<0.001
Positive	6.28	0.98	6.16	1.18	Main effect: set	6.537	0.029
Neutral	1.88	0.36	1.75	0.36	Interaction effect: emotion*set	0.002	0.969

A 2 emotion (positive, neutral) × 2 set (Set1, Set2) two-way ANOVA was performed on the valence and arousal ratings of the scene stimuli. A main effect of emotion was observed in both the valence and arousal ratings with the positive scenes being rated higher in both valence and arousal than the neutral scenes. No emotion × set interaction was observed for both valence and arousal ratings.

### MRI data collection

The MRI data were collected using a 32-channel head coil on the 3 T Siemens Skyra MRI scanner at the HCG Hospital. During functional scans, 41 interleaved slices were acquired per volume using the echoplanar imaging (EPI) scan sequence (TR, 2,500 ms; TE, 28 ms; slice thickness, 3 mm; in-plane resolution, 3 mm × 3 mm; voxel size, 3 mm isotropic; flip angle, 79°). The functional slices were positioned at ∼20° oblique orientation clockwise relative to the line connecting the anterior and posterior commissures to minimize susceptibility artifacts in key regions of interest (ROIs) such as the vmPFC and amygdala. In each of the four main experimental task runs, 157 volumes were acquired, which included a 5 s blank screen at the start to account for MR equilibration effects and a 10 s blank screen at the end of the run to capture the hemodynamic response of the last trial. Finally, before the start of the functional EPI scans, a T1-weighted whole-brain structural scan was collected using the MPRAGE sequence (TR, 2,300 ms; TE, 1.99 ms; in-plane field of view, 256 mm; voxel size, 1 mm isotropic; T1, 900 ms; flip angle, 9°).

### MRI preprocessing

First, the raw Digital Imaging and Communications in Medicine files from the structural and functional EPI scans were converted into Neuroimaging Informatics Technology Initiative (NIFTI) format using the Dimon tool from the Analysis of Functional NeuroImages (AFNI; Cox, 1996). Before converting to NIFTI, the first two volumes from each functional scan were excluded to account for MR equilibration effects.

The anatomical images were bias field corrected using *N4BiasFieldCorrection* from Advanced Normalization Tools (ANTs; Avants et al., 2011). Skull stripping was performed using a consensus-based voting scheme by employing algorithms from the following software packages: (1) *antsBrainExtraction.sh* from ANTs, (2) ROBEX (Iglesias et al., 2011), (3) *bet* from FMRIB Software Library (FSL) (Jenkinson et al., 2012), (4) *3dSkullStrip* from AFNI (Cox, 1996), (5) *mri_watershed* from FreeSurfer (Fischl, 2012), (6) *bse* from BrainSuite (Shattuck and Leahy, 2002), and (7) spm_segment from Statistical Parametric Mapping (Ashburner and Friston, 2005). Voxels that were classified as in-brain by at least six out of seven packages were considered to be part of the final skull-stripped anatomical image [see Meyer et al. (2019) for a similar strategy]. The skull-stripped anatomical image was then rotated to the oblique orientation of the functional data (using *3dWarp* from AFNI) and normalized to the skull-stripped MNI152 template at 1 mm resolution using nonlinear registration (using *antsRegistrationSyN.sh* program from ANTs).

The preprocessing of functional data involved the following steps. First, the slice-time correction was performed (using *3dTshift* in AFNI) to align the onset times of every slice in a volume to the first acquisition slice. Then, motion correction was performed (using *3dvolreg* in AFNI) to spatially register all volumes to the first volume, which was closest in time to the high-resolution anatomical image. The first motion-corrected volume was then coregistered with the obliquely oriented anatomical image using boundary-based registration (using the *epi-reg* program from FSL). Then, the motion-corrected data were normalized to the MNI152 template by applying the functional-structural coregistration transform along with the affine and nonlinear transforms from the anatomical normalization in a single step and resampled to 2 mm isotropic voxels (using the *antsApplyTransforms* program from ANTs). Subsequently, the normalized functional data were spatially smoothed using a Gaussian kernel of 6 mm full-width at half-maximum restricted to gray-matter voxels (using *3dBlurInMask* in AFNI). Finally, the mean intensity in each voxel (per run) was scaled to 100 so that the regression estimates from the individual-level GLM analysis (see below, fMRI statistical analysis) can be interpreted in terms of percent signal change.

### fMRI statistical analysis

For the individual-level analysis, the preprocessed time series data in each voxel (restricted to gray-matter voxels) was modeled using multiple linear regression (using *3dDeconvolve* in AFNI) with the following task-related regressors. During the choice stage, two regressors were included for chair and plane choices separately, and during the outcome stage, four regressors were included corresponding to each outcome type (neutral–loss, neutral–no loss, positive–loss, positive–no loss). These six regressors were modeled for 2 s from the corresponding stimulus onset and convolved with a canonical gamma variate hemodynamic response function model (Cohen, 1997). The choice and outcome stage regressors of no-response trials [pooled over all conditions; 1.37 ± 1.61 trials (mean ± SD)], whenever applicable, were also included in the model as regressors of no interest. Additional regressors included in the GLM model were six estimated motion parameters, their derivatives, and polynomial regressors (of 0–3°) separately for each run to account for baseline and drifts of the MR signal. Finally, we excluded volumes [0.58 ± 1.48% (mean ± SD)] with a frame-to-frame displacement of >1.5 mm Euclidean distance from the GLM analysis (using the censor option in 3dDeconvolve).

Our main focus in this study was on the outcome stage of the task. Hence, for the group-level whole-brain voxel-wise analysis, the estimated β coefficients of the four outcome regressors in each voxel (restricted to gray-matter voxels) were subjected to a 2 *Emotion* (positive, neutral) × 2 *Loss* (loss, no-loss) repeated-measures ANOVA (using *3dANOVA3* in AFNI). To account for multiple comparisons, we thresholded the main effects and interaction statistical maps at a voxel-level uncorrected *p* value of 0.001 and a minimum cluster extent of 37 voxels for a cluster-level corrected α of 0.05. The cluster-level threshold was estimated by running 100,000 simulations using the *3dClustSim* program in AFNI restricted to gray-matter voxels. For these simulations, the noise smoothness values in three directions were estimated from the GLM model residual time series in each participant (using the *3dFWHMx* program in AFNI with the recently added “acf” option) and averaged across participants.

### Integration of positive emotion and loss outcomes: interactive versus additive pattern

Our primary research question in this study was to examine the nature of the integration of positive emotion and monetary loss outcomes, whether it would be of interactive or additive pattern. The interactive integration pattern in a brain region would be supported by a significant interaction effect from the 2 *Emotion* (positive, neutral) × 2 *Loss* (loss, no loss) repeated-measures ANOVA analysis. On the other hand, the additive integration pattern expects that both the emotion (positive vs neutral) and loss (loss vs no-loss) manipulations would recruit overlapping brain regions with no interaction between the two. To formally test for the presence of both loss and emotion effects in a brain region, we ran a whole-brain voxel-wise conjunction analysis (Nichols et al., 2005). First, we created two statistical brain masks based on voxels that showed a significant main effect of the emotion factor (at cluster-level corrected α of 0.05) and a significant main effect of the loss factor (at cluster-level corrected α of 0.05). We then created an intersection map of these two masks, which revealed brain regions (a minimum cluster extent of 37 voxels for a cluster-level corrected α of 0.05) commonly activated by the orthogonal emotional and loss manipulations.

### Relationship between monetary loss and positive emotion effects

To further assess the relationship between the effects of monetary loss and positive emotion, we correlated the main effects of emotion and loss factors across participants in a ROI analysis. We conducted this correlational analysis in five clusters that exhibited both main effects in vmPFC, left and right striatum, and left and right amygdala, which are our primary ROIs. Since some of these clusters extended outside our ROIs, we further restricted them in the following way. In the bilateral amygdala, the clusters were restricted to their anatomical definitions based on 50% probabilistic masks from the Harvard–Oxford subcortical structural atlas distributed in FSL. Similarly, the clusters were restricted to the anatomical definitions of the caudate and ventral striatum in the bilateral striatum. Finally, since vmPFC does not refer to a specific anatomical brain region, we restricted the clusters to a functional definition, which was obtained by the intersection of two spheres (6 mm radius) centered on the peak meta-analytic activation coordinates reported for erotic and monetary outcomes (Sescousse et al., 2013).

For each participant, we first averaged the estimated β coefficients of four outcome stage regressors of all voxels in each ROI separately. Then we calculated the main effect index of emotion [(loss + no loss)_Positive_ – (loss + no loss)_Neutral_] and loss [(positive + neutral]_Loss_ – (positive + neutral)_No loss_] factors. Finally, for each ROI, we calculated Pearson’s correlation between these two main effect indices across participants. Accounting for multiple comparisons for the number of ROIs tested, the *p* value was set at 0.01 for these analyses (using the Bonferroni’s correction) for an overall α value of 0.05.

### Subjective ratings analysis

To probe for potential modulatory effects of positive emotion on the subjective experience of loss outcomes, a 2 *Emotion* (positive, neutral) × 2 *Loss* (loss, no-loss) repeated-measures ANOVA was conducted (using JASP software; Love et al., 2019) on the subjective ratings collected for each of the four outcome conditions at the end of two phases. The *p* value was set at 0.05 for this analysis.

### Behavioral choice analysis

Since participants were instructed that their choice determines the monetary outcome, the proportion of chair and plane choices was calculated separately during each phase of the main experiment. As noted previously, in one phase (Phase 1), positive and neutral stimuli signaled loss and no-loss outcomes, respectively, and in the other phase (Phase 2), this valence–outcome mapping was reversed. Additionally, in each of these phases, the proportion of loss outcome trials following plane and chair choices was computed to confirm that the choices did not differ in their expected loss outcome values. For each metric, the average value across participants was statistically compared against the chance value (50%) using a one-sample *t* test. The *p* value was set at 0.05 for this analysis.

### Learning effects during the choice task

In our choice task, as participants were asked to actively choose between chair and plane stimuli to minimize their monetary losses, they may have attempted to learn the associations between their actions and outcomes. Interestingly, Chien et al. (2016) investigated the effects of inherent stimulus values on learning action–outcome associations using a reinforcement learning (RL) model. During the choice task, the authors employed facial stimuli with two levels of inherent value (based on attractiveness ratings), high and low, each associated with two levels of reward outcome probability: high (70%) and low (30%). The authors found that learning was faster when the inherent values of a stimulus and outcome probability were both high and low (value congruence conditions) than when the inherent value and outcome probability did not match (value incongruence conditions). Unlike the Chien et al. (2016) study, our choice task involved images of chairs and planes with little inherent value. Despite this, participants may have preferred to choose one stimulus over the other, assigning it a higher inherent value.

Therefore, to estimate if there were differential learning effects for the two apparently neutral stimuli in our choice task, chairs and planes, we employed the classic RL algorithm (Rescorla and Wagner, 1972; Sutton and Barto, 1998) to model each participant's choice behavior. The expected value of each stimulus at every trial *t* was represented by Vt. For the subsequent trial *t + 1*, the expected value of the chosen option was updated (Eq. 1) in proportion to the prediction error (PE) on trial *t* (i.e., the difference between the actual outcome Rt and expected outcome *Vt*; Eq. 2) and the learning rate alpha (α), while the value of the nonchosen option remained unchanged:

(1) V(t+1)=Vt+α*PE,

(2) PE=Rt−Vt.

The updated values were then utilized by the model to make decisions on the subsequent trial. This was facilitated by using the softmax choice probability function, which converted the expected values to choice probabilities (for instance, *V*(Chair) was converted to *p*(chair) after passing through the softmax equation; Eq. 3). The higher the *p*(chair) compared with *p*(plane), the more likely chair stimulus would be selected on the subsequent trial:

(3) $$p(\mathrm{chair})=\frac{\exp\;(\beta *V(\mathrm{chair}))}{\exp\;(\beta *V(\mathrm{chair}))+\exp\;(\beta *V(\mathrm{plane}))},$$

where the probability of choosing the chair stimulus, *p*(chair), was determined by the softmax equation as a function of the expected values of two stimuli and the inverse temperature parameter beta (β). β represents choice consistency—a larger β implies that participants are more likely to pick the stimulus with the higher expected value, or in other words, choices become more deterministic as the expected value difference between stimuli increases. A smaller β indicates a more exploratory behavior between the available stimulus options and the choice probability gets closer to 0.5, irrespective of the difference in expected values.

While the learning rate (α) and the inverse temperature (β) are the two key parameters forming the basis of an RL model, we also included the initial expected values for the chair (V0_Chair_) and plane (V0_Plane_) stimuli as free parameters, which index a measure of initial bias or preference for each stimulus [see Chien et al. (2016) for a similar strategy]. We used grid search to estimate the combination of parameter values that maximizes the probability of the observed behavior. Specifically, the RL model was run for the whole range of values for α_Chair_ (0–1), α_Plane_ (0–1), β_Chair_ (0–10), β_Plane_ (0–10), V0_Chair_ (0–1), and V0_Plane_ (0–1) parameters, to calculate the mean square error (MSE) between the model prediction and the observed choice behavior (coded as a 1 for chair choice and a 0 for plane choice) for each set of parameter values (Eq. 4). Finally, the parameter values of the RL model, which resulted in a minimum MSE, were considered the best-fit parameter values:

(4) $$\mathrm{MSE}=\mathrm{mean}[(\mathrm{observed}\;\mathrm{choice}-p(\mathrm{chair}{)}^{2}]\;.$$

In each participant, the RL model of choice behavior was done separately for each of the two experimental phases, one where positive images signaled a loss outcome and neutral ones signaled a no-loss outcome (Phase 1) and the other in which positive images signaled a no-loss outcome and neutral images signaled a loss outcome (Phase 2). Hence, two learning rates (α_Chair_ and α_Plane_), corresponding to the two stimuli, were estimated separately for each of the two phases in each participant.

Firstly, we examined if there is any evidence for learning associations between actions and outcomes in our choice task. To do so, for each phase separately, we compared the estimated learning rates of chair and plane stimuli against zero using a one-sample *t* test. Secondly, across the participants, if one stimulus was consistently assigned a higher inherent value compared with the other, then the learning rates of the two stimuli were expected to differ. To test this, in each phase separately, we compared the estimated learning rates of chair and plane stimuli using a paired *t* test. Finally, it is plausible that some participants might have assigned a higher inherent value for one stimulus (say for chair over the plane), whereas the others might have assigned a higher inherent value for plane over the chair stimulus. According to Chien et al (2016), stimuli with higher inherent value (more attractive facial images in their study) led to a higher frequency of selection. So, for each participant, based on the proportion of chair and plane choices made in each phase (Table 7), we presumed that the stimulus that was selected more often might have been assigned a higher inherent value. If our assumption is valid, based on the findings of Chien et al (2016), we expected that the learning rate for deemed higher inherent value stimulus (could be chair or plane, depending on the participant) would be greater than that of the (relatively) lower inherent value stimulus. To test this, separately in each phase, the learning rates of the deemed higher and lower inherent value stimulus were compared using a paired *t* test. The *p* value was set at 0.05 for the statistical analyses involving learning rates. Additionally, in the cases of nonsignificant results, we performed the corresponding *t* tests in a Bayesian framework (Wagenmakers et al., 2018) to calculate the inverse Bayes factors (BF01), which indicate the likelihood of the evidence in favor of the null hypothesis.

## Results

### fMRI

**Figure 2.**
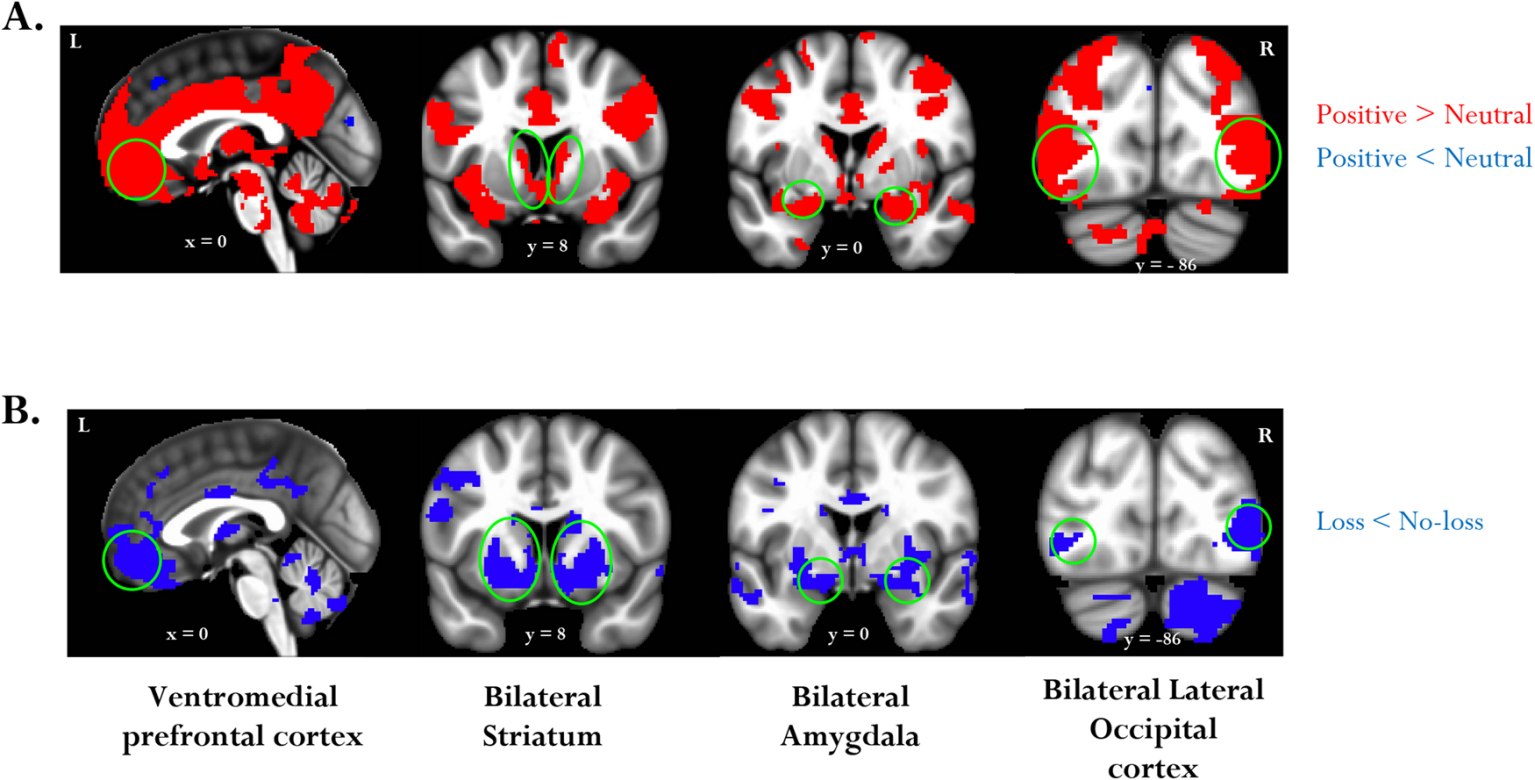
Main effects of emotion and loss. Clusters that exhibited the main effects of (***A***) *Emotion* and (***B***) *Loss* outcomes at an uncorrected *p* = 0.001 and 37-voxel cluster extent (cluster-level α of 0.05). Because cluster-extent–based thresholding was used for multiple-comparison correction, voxels are displayed using a binary threshold. For further rationale about using binary maps in the context of cluster-based thresholding, see Woo et al., 2014.

Our primary research question in this study was to examine the nature of the integration of positive emotion and monetary loss outcomes. To do so, in a whole-brain voxel-wise analysis, we ran a 2 *Emotion* (positive, neutral) × 2 *Loss* (loss, no-loss) repeated-measures ANOVA using β estimates from the outcome stage. After the cluster-level correction for multiple comparisons, we observed a significant main effect of emotion in several regions, including the vmPFC, bilateral striatum, bilateral amygdala, cingulate cortex, and bilateral lateral occipital cortex (Fig. 2*A*; Table 2). In all these clusters, positive images evoked higher activation than neutral ones. We also observed a significant main effect of loss after the cluster-level correction in multiple regions, including the vmPFC, bilateral striatum, bilateral amygdala, and bilateral lateral occipital cortex (Fig. 2*B*; Table 3). In all these clusters, loss outcomes evoked lower activation than no-loss outcomes. Crucially, we did not detect any regions with significant interaction effects after the cluster-level correction, indicating a lack of evidence supporting the interactive pattern of integration between positive emotion and loss outcomes.

**Table 2.** Main effect of *Emotion* outcome

Positive > neutral ES = [(loss + no loss)_Positive_ – (loss + no loss)_Neutral_]						
Cluster	*x*	*y*	*z*	*k*	*F*_(1,34)_	ES
Medial frontal cortex, cingulate cortex, precuneus, fusiform gyrus, cerebellum, thalamus, brainstem, midbrain	56	−72	−4	42,267	50.69	1.36
R. precentral gyrus	50	6	36	1,622	47.14	0.46
L. precentral gyrus	−50	0	34	1,119	23.02	0.26
R. inferior frontal gyrus	54	40	10	231	26.93	0.34
R. middle temporal gyrus	64	0	−22	177	14.03	0.14
L. anterior insula	−40	26	4	146	60.66	0.14
L. inferior frontal gyrus	−52	40	10	146	20.17	0.19
R. superior frontal gyrus	2	10	62	124	14.69	0.11
L. perirhinal/rhinal sulcus	−38	−4	−34	111	25.33	0.16
L. middle insula	−36	−6	18	61	35.66	0.11
R. globus pallidus	22	−2	8	53	14.45	0.06
R. middle insula	38	−2	14	38	27.44	0.12
Neutral > positive ES, [(loss + no loss)_Neutral_ – (loss + no loss)_Positive_]						
Brain regions	*x*	*y*	*z*	*k*	*F*_(1,34)_	ES
R. parahippocampal gyrus	28	−46	−10	537	168.18	0.30
L. parahippocampal gyrus	−28	−60	−10	479	70.27	0.25
R. angular gyrus	52	−60	50	433	17.17	0.18
R. medial frontal gyrus, inferior frontal sulcus	42	26	40	336	24.26	0.16
L. anterior transverse temporal gyrus	−56	−16	10	271	18.33	0.14
R. superior temporal gyrus	68	−10	−6	196	14.59	0.18
R. superior frontal gyrus	4	28	44	155	37.62	0.11
R. transverse temporal gyrus	50	−28	14	110	19.21	0.14
R. superior/middle temporal gyrus	−46	−32	8	64	20.73	0.11
Calcarine sulcus/striate cortex	0	−84	22	37	13.24	0.11

The following clusters survived multiple-comparison correction in univariate whole-brain voxel-wise analysis at the outcome phase [peak MNI coordinates, cluster size (k), *F*_(1,34)_ values, and unstandardized effect size in terms of % signal change (ES)].

**Table 3.** Main effect of *Loss* outcome

No loss > loss ES = [(positive + neutral)_No loss_ – (positive + neutral)_Loss_]						
Cluster	*x*	*y*	*z*	*k*	*F*_(1,34)_	ES
Bilateral striatum	−6	0	4	3,936	15.23	0.35
Medial frontal cortex (vmPFC)	0	62	4	2,385	31.57	0.24
R. cerebellum (lateral zone)	48	−72	−42	2,158	40.87	0.29
R. middle/inferior temporal gyrus	60	−56	−16	1,171	14.47	0.20
R. inferior occipital gyrus	26	−98	−12	863	19.52	0.29
L. middle temporal gyrus	−64	−54	−8	791	13.58	0.19
L. inferior occipital gyrus	−6	−98	−18	763	13.31	0.32
L. middle/inferior frontal gyrus	−50	40	18	693	15.05	0.15
L. cerebellum (lateral zone)	−42	−74	−48	658	16.67	0.21
R. posterior cingulate cortex/precuneus	0	−46	38	459	14.06	0.13
R. anterior orbital gyrus	40	40	−20	394	16.50	0.19
Cerebellum (vermis)	−2	−42	−36	354	14.16	0.17
L. precentral gyrus, inferior precentral sulcus	−54	8	42	223	15.03	0.19
R. superior frontal gyrus (lateral part), accessory superior frontal sulcus	26	30	52	204	22.08	0.09
Midcingulate cortex	0	−8	28	155	15.53	0.12
L. cerebellum (posterior lobe)	−20	−86	−46	144	13.31	0.22
L. lateral posterior Orbital gyrus, sulcus	−20	36	−22	133	13.33	0.14
L. inferior frontal gyrus	−60	12	22	129	16.33	0.12
L. superior frontal gyrus, paracingulate	0	30	42	120	14.05	0.13
L. angular gyrus	−50	−70	42	106	13.29	0.16
L. cerebellum (anterior lobe)	−10	−84	−24	105	14.40	0.13
Cerebellum (posterior lobe, vermis)	4	−58	−22	100	17.58	0.09
L. superior temporal gyrus	−68	−28	14	90	14.48	0.18
L. paracentral lobule	−2	−24	58	83	14.28	0.10
L. superior frontal gyrus, accessory superior frontal sulcus	−22	32	56	80	14.66	0.12
L. cerebellum (anterior lobule)	−10	−36	−16	75	13.13	0.11
R. cerebellum (anterior lobule)	18	−28	−28	69	13.48	0.11
Cerebellum (central lobule)	−4	−44	−8	61	20.10	0.09
L. striate cortex	−2	−102	12	60	13.41	0.19
L. supramarginal gyrus/superior parietal lobule	−34	−44	38	57	18.34	0.08
L. middle temporal gyrus, superior temporal sulcus	−58	−38	0	46	13.77	0.08
R. paracentral lobule	4	−28	58	40	14.26	0.10
L. precentral gyrus	−10	−30	66	39	27.56	0.08
L. lateral caudate	−18	−6	26	38	21.48	0.18
Loss > no loss ES = [(positive + neutral)_Loss_–(positive + neutral)_No Loss_]						
No clusters found.						

The following clusters survived multiple-comparison correction in univariate voxel-wise analysis at the outcome phase [peak MNI coordinates, cluster size (k), *F*_(1,34)_ values, and unstandardized effect size in terms of % signal change (ES)].

**Figure 3.**
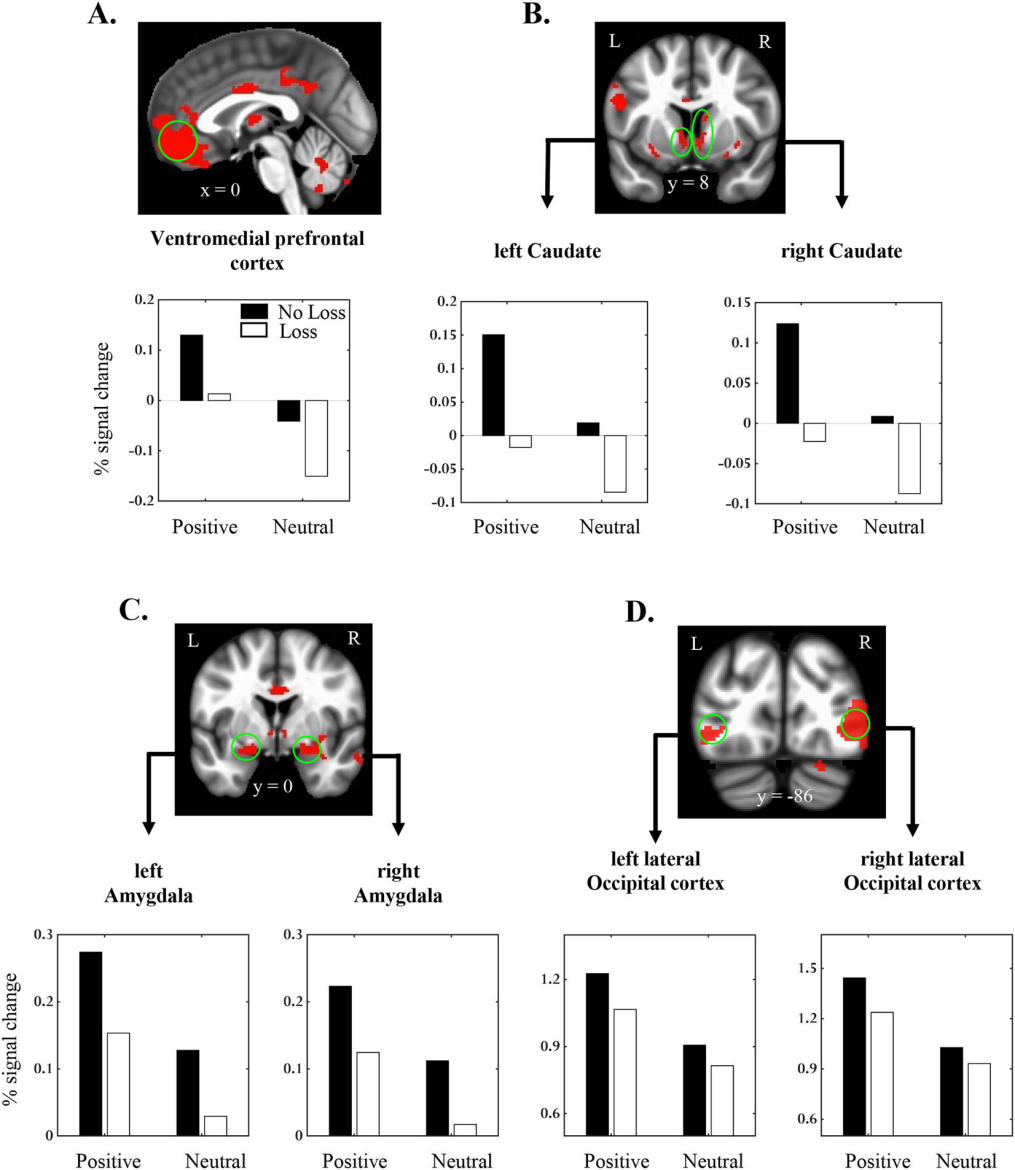
Conjunction of emotion and loss effects. Clusters that exhibited conjunction of the main effects of *Emotion* and *Loss* factors in (***A***) vmPFC, (***B***) bilateral caudate, (***C***) bilateral amygdala, and (***D***) bilateral lateral occipital cortex. For each cluster, the bar plot shows the average group-level estimates of the four outcome conditions of interest. It should be noted that the primary purpose of these average bar plots is to illustrate the overlapping pattern of the main effects and not to draw any statistical inference. Hence, error bars were not plotted on top of the average estimates to avoid issues of circularity (Kriegeskorte et al., 2009).

Next, to identify the common brain regions recruited by both emotion and loss manipulations, we performed a conjunction analysis based on the significant main effects in the whole-brain voxel-wise analysis. The conjunction analysis revealed overlapping main effects in multiple regions, notably, vmPFC, bilateral striatum (encompassing caudate and ventral striatum), bilateral amygdala, and bilateral lateral occipital cortex (Fig. 3; Table 4). The presence of the two main effects with a lack of evidence for the significant interaction in these regions suggests that the effects of positive emotion and loss outcomes were additive in nature. Additionally, in the clusters that exhibited overlapping main effects, we performed 2 *Emotion* (positive, neutral) × 2 *Loss* (loss, no-loss) repeated-measures ANOVA in a Bayesian framework (Wagenmakers et al., 2018) to calculate the likelihood of the absence of the interaction effect (i.e., evidence in favor of the null hypothesis). This Bayesian analysis revealed inverse Bayes factors (BF01) of >3.5 in multiple regions of our primary interest including the vmPFC and bilateral amygdala (Table 4), indicating “moderate” evidence for the null interaction effects.

**Table 4.** Conjunction of main effects of *Emotion* and *Loss*

Cluster	*x*	*y*	*z*	*k*	BF01
vmPFC	2	26	−28	1,895	4.39
R. lateral occipital cortex	30	−92	−14	764	1.23
R. cerebellum (lateral zone)	18	−76	−46	562	3.05
R. amygdala	32	−4	−22	486	3.87
L. lateral occipital cortex	−32	−96	−14	451	1.70
L. amygdala	−26	−4	−20	241	3.51
L. posterior Cingulate cortex	−8	−54	20	212	2.21
R. lateral Orbitofrontal cortex	30	36	−20	169	3.90
Midcingulate cortex	−2	6	26	141	2.32
R. inferior temporal gyrus/fusiform gyrus	46	−50	−20	123	2.83
Cerebellum (posterior lateral fissure)	−10	−46	−48	120	1.66
L. lateral Orbitofrontal cortex	−20	28	−18	120	2.36
R. caudate/ventral Striatum	6	8	−10	113	1.53
Inferior thalamic peduncle	6	−4	2	94	1.28
L. intermediate Frontal sulcus	−52	36	4	83	1.28
L. caudate/ventral Striatum	−4	4	−8	80	1.08
L. inferior frontal gyrus	−52	6	20	69	3.94
Cerebellum (vermis)	0	−60	−32	60	4.09
L. cerebellum (lateral zone)	−30	−74	−42	53	2.44
L. cerebellum (intermediate zone)	−6	−80	−49	52	2.11
R. posterior cingulate cortex	8	−52	18	49	2.04
L. inferior precentral sulcus	−42	4	36	49	1.33
R. middle temporal gyrus	62	−2	−24	40	4.30
L. supramarginal gyrus	−34	−44	34	39	2.32

The following clusters survived the multiple-comparison correction in univariate voxel-wise conjunction analysis at the outcome phase [MNI coordinates, cluster size (k), inverse Bayes factors (BF01) values indicating the evidence in favor of null interaction effects].

**Figure 4.**
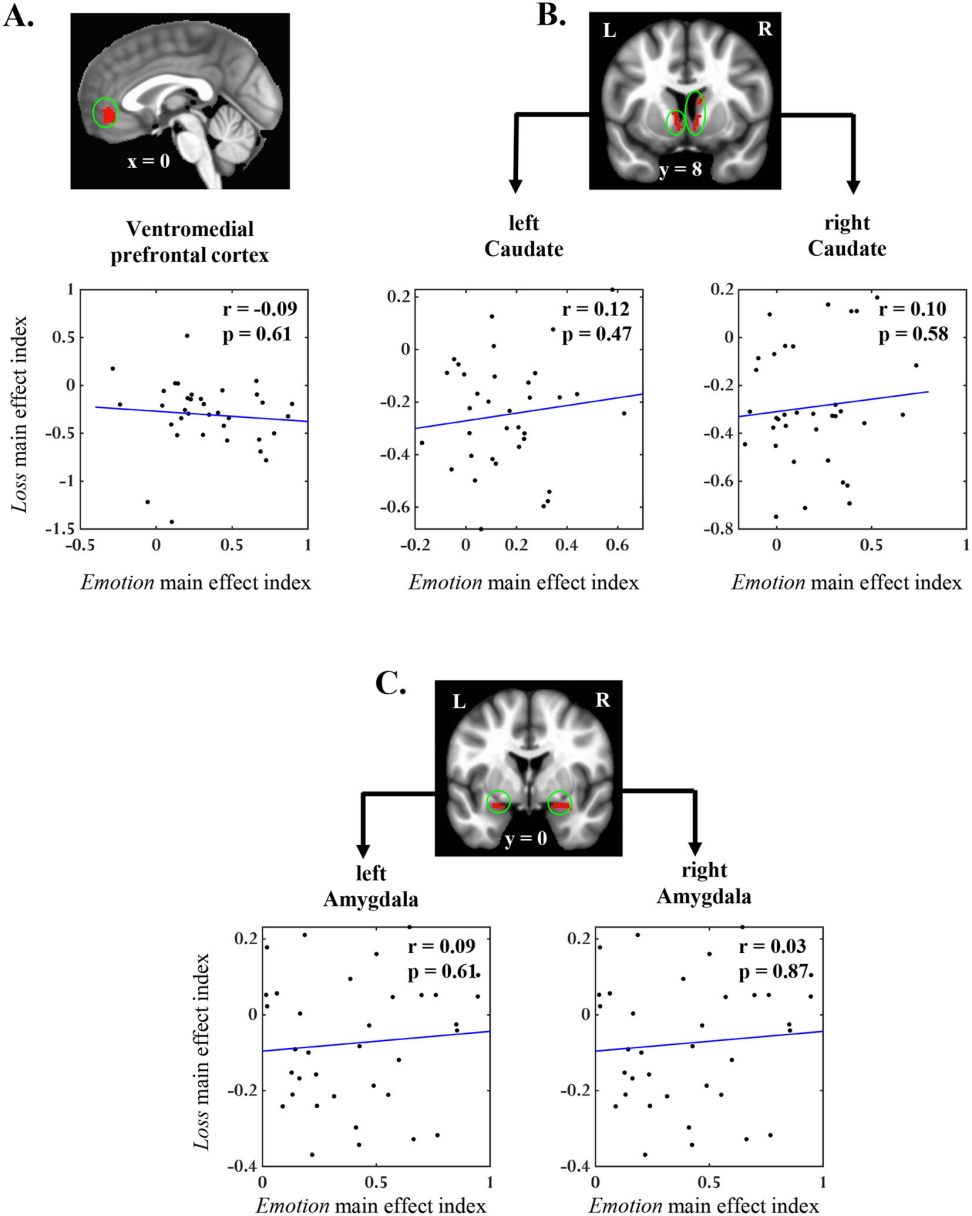
Relationship between emotion and loss effects. Pearson's correlation between *Emotion* and *Loss* main effects across participants in clusters that exhibited conjunction of the main effects in (***A***) vmPFC, (***B***) bilateral caudate, and (***C***) bilateral amygdala. *Emotion* main effect index: (loss + no loss)_Positive_ – (loss + no loss)_Neutral_; *Loss* main effect index: (positive + neutral)_Loss_ – (positive + neutral)_No loss_.

To further assess the relationship between monetary loss and positive emotion in clusters that exhibited both main effects, we correlated the main effects of emotion and loss factors across participants in an ROI analysis (see Materials and Methods, Relationship between monetary loss and positive emotion effects). In none of the five ROIs, we observed a significant correlation between the two main effects (Fig. 4; Table 5), suggesting that the influences of monetary loss and positive emotion were largely independent in regions of our primary interest. Furthermore, using JASP, we performed the same correlation analysis in a Bayesian framework (Wagenmakers et al., 2018) to calculate the likelihood of the absence of the correlation effect (i.e., evidence in favor of the null hypothesis). This Bayesian analysis revealed inverse Bayes factors (BF01) of >4.0 in multiple ROIs (Table 5), indicating “moderate” evidence for the null correlation effects.

**Table 5.** Relationship between *Emotion* and *Loss* effects

Brain region	*x*	*y*	*z*	*k*	*r*_(33)_	*p* value	BF01
vmPFC	0	40	−12	110	−0.088	0.614	4.21
R. amygdala	32	4	−22	175	0.028	0.875	4.69
L. amygdala	−26	−4	−20	118	0.088	0.613	4.21
R. caudate/ventral striatum	6	−8	−8	69	0.153	0.379	4.12
L. caudate/ventral striatum	−6	−6	−8	52	0.168	0.336	3.72

Correlation between emotion and loss main effects in clusters of our primary ROIs (MNI coordinates, cluster size (k), Pearson's correlation (*r*) and the corresponding *p* value, inverse Bayes factors (BF01) values indicating the evidence in favor of null correlation effects).

### Subjective ratings

**Figure 5.**
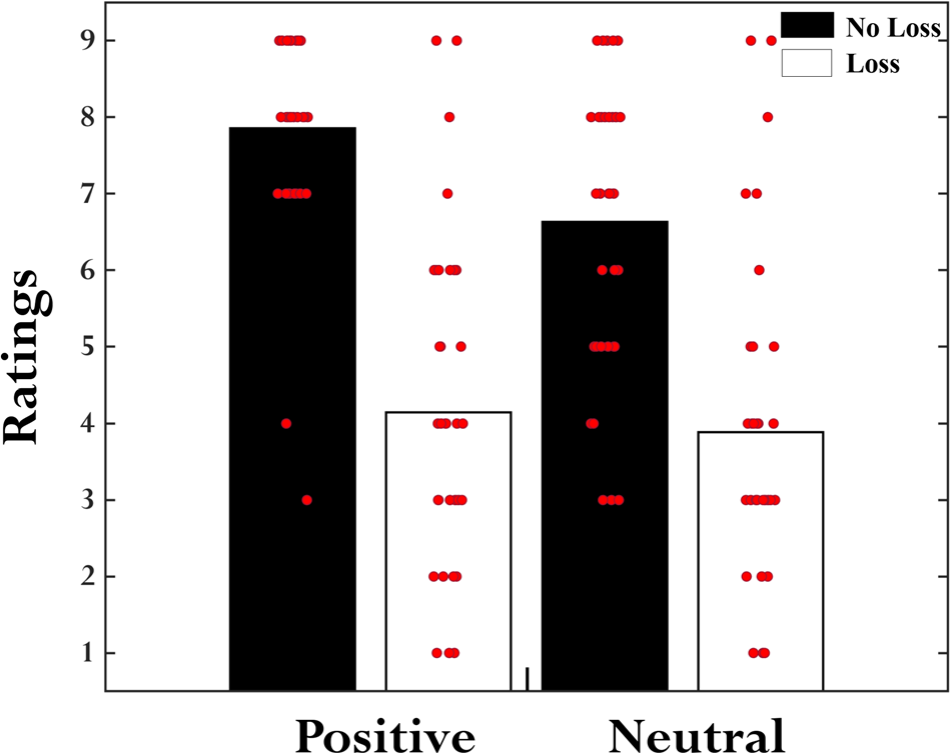
Subjective hedonic ratings of experienced outcomes. The bar plot shows the average self-reported pleasantness ratings for the four outcome conditions on a scale of 1–9 (1 being unpleasant and 9 being pleasant). Each dot indicates the individual participant ratings.

A 2 *Emotion* (positive, neutral) × 2 *Loss* (loss, no-loss) repeated-measures ANOVA on the subjective pleasantness ratings collected at the end of each phase (Fig. 5; Table 6) revealed the main effects of both *Emotion* (*F*_(1,34)_ = 9.09; *p* = 0.005) and *Loss* (*F*_(1,34)_ = 90.1; *p* < 0.001) factors. The positive images (mean, 6.0; SD, 2.57) were rated as more pleasant than neutral ones (mean, 5.25; SD, 2.38), and loss outcomes (mean, 4.01; SD, 2.08) were rated as less pleasant than no-loss outcomes (mean, 7.24; SD, 1.72). These observed patterns of main effects in subjective ratings validate our positive emotion and monetary loss manipulations. However, the interaction between the two factors was not detected (*F*_(1,34)_ = 2.77; *p* = 0.105). Additionally, using JASP, we performed 2 *Emotion* (positive, neutral) × 2 *Loss* (loss, no-loss) repeated-measures ANOVA on the subjective ratings in a Bayesian framework (Wagenmakers et al., 2018) to calculate the likelihood of the absence of the interaction effect (i.e., evidence in favor of the null hypothesis). This Bayesian analysis revealed inverse Bayes factor (BF01) of 0.93, indicating anecdotal evidence for the null interaction effects.

**Table 6.** Subjective ratings

Outcome condition	Mean ± SD
Please rate how you felt when the NO-LOSS information was signaled by a POSITIVE image	7.85 ± 1.33
Please rate how you felt when the LOSS information was signaled by a POSITIVE image	4.41 ± 2.14
Please rate how you felt when the NO-LOSS information was signaled by a NEUTRAL image	6.63 ± 1.86
Please rate how you felt when the LOSS information was signaled by a NEUTRAL image	3.89 ± 2.05

Average self-reported pleasantness ratings for the four outcome conditions on a scale of 1–9 (1 being unpleasant and 9 being pleasant), collected at the end of each phase.

### Choice behavior

Across both phases, participants exhibited a high response rate (98.63 ± 1.61%), indicating they were attentive to the choice task. In Phase 1, where positive images signaled loss outcomes and neutral ones signaled no-loss outcomes, the proportions of the chair and the plane choices did not differ from 50% across the participants (Table 7). However, in Phase 2, where neutral images signaled loss outcomes and positive ones signaled no-loss outcomes, the proportion of the chair and the plane choices differed from 50% across the participants (Table 7), indicating some preference to choose one stimulus over the other. Furthermore, we also computed the proportions of loss outcomes following chair and plane choices in both phases and found them not to significantly differ from the chance level (Table 7). This result indicates that despite the predetermined outcomes, the loss versus no-loss outcome expectancies for the two choices were not skewed in favor of one over the other.

**Table 7.** Behavioral choice measures separately for two phases

Conditions	Phase 1			Phase 2		
	Mean ± SD (%)	*t*_(34)_	*p* value	Mean ± SD (%)	*t*_(34)_	*p* value
Chair choices	49.29 ± 13.69	−0.31	0.76	42.62 ± 14.27	−2.06	0.004
Plane choices	49.37 ± 13.83	−0.27	0.79	55.99 ± 14.59	2.43	0.021
Chair choices that led to Loss outcome	51.03 ± 5.26	1.15	0.26	53.28 ± 10.21	1.90	0.07
Plane choices that led to Loss outcome	48.86 ± 5.59	−1.20	0.24	48.51 ± 6.69	−1.31	0.19

In Phase 1, positive stimulus signaled a loss outcome and neutral stimulus signaled a no-loss outcome. In Phase 2, neutral stimulus signaled a loss outcome, and positive stimulus signaled a no-loss outcome. The *t*_(34)_ and corresponding *p* values are based on a one-sample *t* test against the chance value (50%).

#### Learning effects during the choice task

**Figure 6.**
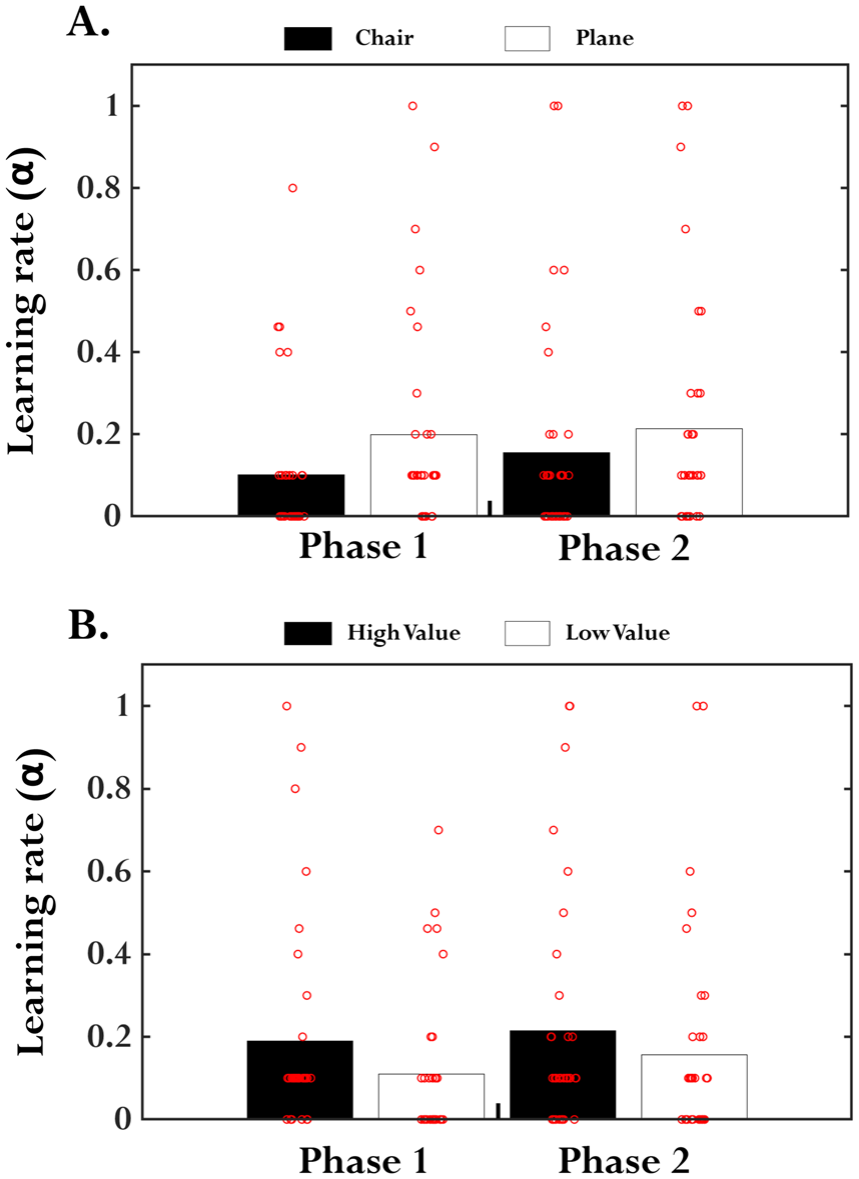
Estimated learning rates from the RL model. ***A***, The bar plot shows the average estimated learning rates for the chair and plane stimulus for two phases separately. In phase ***1***, positive stimulus signaled a loss outcome and neutral stimulus signaled a no-loss outcome. In phase ***2***, neutral stimulus signaled a loss outcome and positive stimulus signaled a no-loss outcome. ***B***, The bar plot shows the average estimated learning rates for the deemed high inherent valued and low inherent valued stimulus for two phases separately. Each dot indicates the estimated learning rate of each participant.

We investigated potential learning effects in our choice task using a classic RL algorithm. In each phase, learning rates of chair and plane stimuli were significantly greater than zero (Table 8; Fig. 6), indicating some degree of learning associations between actions and outcomes in our choice task. However, in each phase, the learning rates of chair and plane stimuli were not significantly different (Phase 1, *t*_(34)_ = −1.8929; *p* = 0.076; BF01, 1.236; Phase 2, *t*_(34)_ = −0.781; *p* = 0.440; BF01, 4.155). Furthermore, in each phase, the learning rates of the deemed higher (could be chair or plane depending on the proportion of choices made by each participant; see Materials and Methods, Learning effects during the choice task) and lower inherent value stimulus were not significantly different (Phase 1, *t*_(34)_ = 1.487; *p* = 0.146; BF01, 2.023; Phase 2, *t*_(34)_ = 0.781; *p* = 0.440; BF01, 4.155). These results suggest that the degree of learning associations between actions and outcomes in our choice task might not have differed between the two apparently neutral stimuli (images of chairs and planes), implying that participants might not have assigned one stimulus a higher inherent value over the other.

**Table 8.** Average estimated learning rates (α) for the different stimulus types, chair and plane and deemed high and low inherent value stimuli for the two phases separately

Stimulus	Phase 1			Phase 2		
	Mean ± SD (%)	*t*_(34)_	*p* value	Mean ± SD (%)	*t*_(34)_	*p* value
Chair	0.101 ± 0.182	3.264	0.003	0.156 ± 0.268	3.466	0.002
Plane	0.199 ± 0.251	4.692	<0.001	0.214 ± 0.284	4.463	<0.001
High value	0.139 ± 0.201	4.382	<0.001	0.216 ± 0.296	4.384	<0.001
Low value	0.161 ± 0.246	3.628	<0.001	0.154 ± 0.255	3.518	<0.001

In Phase 1, positive stimulus signaled a loss outcome and neutral stimulus signaled a no-loss outcome. In Phase 2, neutral stimulus signaled a loss outcome, and positive stimulus signaled a no-loss outcome. The *t*_(34)_ and corresponding *p* values are based on a one-sample *t* test against zero.

## Discussion

In the present fMRI study, we investigated the neural substrates underlying the net experienced value of conflicting outcomes. Specifically, we probed two plausible integration patterns while processing the conflicting value signals involving monetary loss and positive emotion: interactive versus additive. We found overlapping main effects of positive (vs neutral) emotion and loss (vs no-loss) outcomes in multiple brain regions, including the vmPFC, striatum, and amygdala, notably with a lack of evidence for interaction. Thus, our findings revealed the additive integration pattern of monetary loss and positive emotion during the consummatory phase of value processing.

The role of vmPFC in encoding the experienced value has been extensively reported using different types of primary and secondary reinforcers, such as monetary, emotional, and food outcomes (Sescousse et al., 2013). Of relevance to the current study, vmPFC activity proportionally increased with the value of monetary outcomes (i.e., gains > no gain/no loss > losses; Smith et al., 2010; Mowrer et al., 2011; Lin et al., 2012) and with the valence of emotional outcomes (pleasant > neutral > unpleasant; Smith et al., 2010; Lin et al., 2012; Winecoff et al., 2013). These findings suggest that the activity in vmPFC reflects a domain-general experienced value signal underlying the encoding of monetary and emotional outcomes (Sescousse et al., 2013). However, as most of the previous work employed separate manipulations of monetary and emotional outcomes (even in the same set of participants), how the value signals from concurrent monetary and emotional outcomes are integrated in vmPFC is largely unknown. Here, using a novel paradigm where the valence of an emotional image indicated the type of monetary outcome, we examined the nature of the integration of conflicting value signals, specifically involving positive emotion and monetary losses. In line with the past findings mentioned above, we observed the overlapping main effects of loss and emotion in vmPFC with lower activity during the processing of loss (relative to no-loss) outcomes and higher activity during the processing of positive (relative to neutral) emotional images. Importantly, the effects of monetary losses and positive emotion were additive rather than interactive, indicating that the experienced value of loss (vs no-loss) outcomes was the same regardless of the valence of the image that signaled those outcomes. Furthermore, the main effects of loss and emotion factors were uncorrelated across participants, again suggesting that the influences of monetary loss and positive emotion in vmPFC were largely independent.

In addition to vmPFC, the striatum was also frequently reported to be coactivated during the processing of monetary and emotional outcomes (Bartra et al., 2013; Sescousse et al., 2013). Consistent with the previously reported findings based on separate emotional and monetary outcome manipulations (Sabatinelli et al., 2007; Sescousse et al., 2010; Smith et al., 2010; Mowrer et al., 2011), we observed the main effects of both emotion (positive > neutral) and loss (loss < no-loss) factors in clusters encompassing bilateral caudate and ventral striatum. Although the additive integration patterns observed in vmPFC and striatum are highly similar (Fig. 3), one concern with the interpretation of activity in the striatum is whether it reflects the experienced value (which is typically ascribed to vmPFC) or some other aspects of outcome processing. Even though some earlier studies have interpreted striatum activity during outcome processing in terms of value coding (Delgado et al., 2000), a later set of studies have attributed the role of striatum's activity in encoding prediction errors that signal the discrepancy between received and expected outcomes rather than the experienced value per se (O’Doherty, 2004; Hare et al., 2008; Rutledge et al., 2010). Our current design did not allow us to tease apart the value signals from prediction errors, as positive and negative prediction errors of equal magnitude were expected to be generated during no-loss and loss outcomes, respectively. This is because the proportion of loss and no-loss outcomes experienced after the two types of choices was ∼50% (Table 7), indicating that the expectancies for both outcomes were similar. Hence, the current study cannot provide unequivocal evidence regarding the integration of conflicting value signals in the striatum.

Besides vmPFC and striatum, we also observed the overlapping main effects of our positive emotion and monetary loss manipulations in the amygdala. Previous work has implicated the amygdala in processing salient emotional and monetary outcomes (Liu et al., 2011; Sabatinelli et al., 2011; Sescousse et al., 2013; Oldham et al., 2018). Here, in line with this meta-analytic evidence, we observed higher activity during the processing of positive emotional images than neutral ones in the bilateral amygdala. In the same clusters, we also observed a strong main effect of loss, with lower activation for loss outcomes than no-loss outcomes. The direction of the main effect of loss in the amygdala suggests that at least in the context of our paradigm where only loss and no-loss outcomes were involved, no-loss outcomes might have been perceived as more salient because they were favorable ones and directly relevant to the participants' behavioral goal, which was to minimize monetary losses. This interpretation is in line with proposals that postulated the role of goal relevance in shaping the amygdala responses (Cunningham et al., 2008; Cunningham and Brosch, 2012). Finally, the additive integration pattern observed in the amygdala might reflect the overall saliency of the compound emotional and monetary outcomes, with positive emotion signaling no-loss outcome being the most salient.

Outside our regions of primary interest, clusters exhibiting overlapping main effects were detected in a few other brain regions (Table 4). In particular, the activity patterns observed in the bilateral lateral occipital cortex are worth mentioning. In the lateral occipital cortex clusters, higher activation was observed while processing salient positive emotion relative to neutral images, consistent with the meta-analytic findings of emotional scene perception (Sabatinelli et al., 2011). The enhanced activity observed during erotic (vs neutral) images in the lateral occipital cortex could also be attributed to the differences in the complexity of visual features. There is evidence that information regarding category-specific visual features encoded in the human visual cortex could be reliably mapped to distinct emotions (Kragel et al., 2019; Bo et al., 2021). In the same lateral occipital cortex clusters, we also found the main effect of loss, with activity being lower during loss compared with no-loss outcomes. This main effect is noteworthy given that only positive and neutral emotional images were presented during the outcome phase and participants had to decipher whether they signaled monetary loss or no-loss outcome. This finding indicates that in addition to the emotional content of images, even the type of monetary outcome they signaled influenced the activity in the lateral occipital cortex, giving rise to an additive integration pattern of monetary loss and positive emotion.

The additive effects of positive emotion and monetary losses observed in multiple brain regions were also reflected in the subjective ratings data. The main effect of emotion indicated that positive feedback images were rated more pleasant compared with neutral ones, and the main effect of loss indicated loss outcomes were rated as less pleasant than no-loss outcomes. Previous studies have reported similar rating patterns but separately for emotional and monetary outcomes (Buchel et al., 2018; Sescousse et al., 2010). Crucially, paralleling the fMRI results, we did not detect a significant *Emotion *× *Loss* interaction in the subjective rating data, suggesting that the effects of positive emotion and monetary losses on subjective ratings were also additive in nature. However, Bayesian analysis indicated only anecdotal evidence for null interaction effects. Of note, unlike previous studies that collected hedonic ratings of experienced outcomes on each trial (Sescousse et al., 2010; Buchel et al., 2018), here we only collected a single retrospective rating score for each of the four emotion–loss pair conditions because of time constraints in our fMRI setup. Hence, our subjective rating findings should be interpreted with caution until further replication.

We observed some evidence for learning associations between actions and outcomes during the choice task, where participants were asked to actively choose between chair and plane stimuli to minimize their monetary losses. However, we did not detect significant differences in learning rates between our chair and plane stimuli, suggesting that one stimulus was not consistently assigned a higher inherent value compared with the other across the participants. Nevertheless, as we observed large between-subject variability in the proportion of choices made (Table 7), we additionally compared the learning rates of deemed higher and lower inherent valued stimulus (could be chair or plane depending on the proportion of choices made by each participant), which also yielded a null result. Overall, unlike in Chien et al. (2016), where the authors had explicitly manipulated the inherent value of choice stimuli, which resulted in differential learning rates, we observed similar learning rates for the two apparently neutral stimuli (images of chairs and planes) employed in our choice task.

As noted in the Introduction, Kim and Anderson (2020) investigated the concurrent processing of conflicting outcomes involving monetary gains and aversive stimulation. The authors concluded that monetary gain and aversive shock outcomes were primarily processed in nonoverlapping brain regions, supporting a modular view of processing the concurrent appetitive and aversive outcomes. For instance, regions like vmPFC and ventral striatum were involved in processing monetary gain outcomes, and the anterior insula and anterior cingulate cortex were sensitive to aversive shock outcomes. These findings do not align with the ones we report here, where multiple brain regions exhibited overlapping main effects, revealing their sensitivity to emotional and loss manipulations. Unlike in the design employed by Kim and Anderson (2020) where separate reward and aversive outcomes were presented concurrently, our novel design mandated processing of the valence (positive vs neutral) of an emotional image to determine the type of outcome (loss or no loss), thus fostering the integrated processing of conflicting value signals. In particular, a strong main effect of loss observed in the current study indicates that participants successfully learned the instructed emotion–loss mapping to infer whether their choice led to a loss or no-loss outcome based on the valence of the emotional image. Interestingly, even though both emotional and loss manipulations engaged common brain regions, their effects seem largely independent, that is, additive in nature.

Among the different patterns of integration proposed during the processing of conflicting value signals (Talmi and Pine, 2012), some previous fMRI studies that specifically focused on the decision-making or reward motivation paradigms (Talmi et al., 2009; Park et al. 2011; Choi et al. 2014; Aupperle et al. 2015) reported competitive interaction patterns between monetary rewards and aversive information. The findings from these studies demonstrated that a wide array of aversive manipulations (e.g., pain, aversive images, threat-of-shock) attenuated the anticipatory reward value signals in multiple brain regions, including the striatum and subgenual anterior cingulate cortex. On the other hand, in another fMRI study involving a cognitive control task, Braver and colleagues reported additive integration pattern during anticipation of monetary rewards and aversive liquids in the dorsal anterior cingulate cortex (Yee et al., 2021). Extending this set of findings from past fMRI studies that specifically focused on the net anticipatory value of conflicting prospective outcomes, our current study revealed the additive nature of integration underlying the net experienced value of conflicting outcomes involving monetary loss and positive emotional images in vmPFC. As mentioned above, to examine the nature of integrating the conflicting value signals, different types of aversive stimuli (pain, aversive images, monetary loss, aversive liquids, etc.) have been employed previously. Despite the differences in their primary/secondary nature, sensory modality, physical attributes, etc., they appear to be similarly engaging the value processing regions such as vmPFC and ventral striatum (Sescousse et al., 2010; Wake and Izuma, 2017; Oren et al., 2022), supporting the hypothesis of a neural common currency (Levy and Glimcher, 2012; Sescousse et al., 2013). Based on this notion of common value representation and our current findings, aversive outcomes (regardless of the type) are expected to integrate with positive outcomes in an additive manner, which could be examined in future work.

Contrary to the additive effects we observed in the current study, one previous event-related potential (ERP) study reported an interaction between positive emotion and monetary loss outcomes (Bandyopadhyay et al., 2019). The authors examined the impact of concurrent emotional scene stimuli on monetary gain and loss outcome processing in a gambling task. The ERP analysis was focused on the feedback-related negativity (FRN) component, which is sensitive to outcome evaluation (Gehring and Willoughby, 2002; San Martín, 2012). The FRN response during the processing of loss (vs gain) outcomes was larger in the presence of positive images than neutral ones, supporting the interactive integration. Of note, one key difference exists between the emotional manipulations of the ERP study and the present study. In the ERP study (Bandyopadhyay et al., 2019), emotional scene images were presented as background pictures, on top of which monetary outcome information was displayed, rendering the emotional manipulation task irrelevant. In contrast, emotional manipulation was task relevant in the current study as participants had to process the valence of the feedback image to ascertain the type of monetary outcome they received. Hence, future work could systematically examine the role of the task relevance of emotional information when integrating conflicting outcomes.

Some limitations of the current study are worth mentioning. Here, we focused on investigating the nature of the integration of conflicting outcomes entailing positive emotion and monetary losses using a 2 emotion (positive, neutral) × 2 loss (loss, no-loss) within-subject design. Since the design is not fully crossed, we cannot rule out the potential differential valuation of a particular outcome type in relation to the other outcomes (Breiter et al., 2001; Grabenhorst and Rolls, 2011). For instance, in the absence of monetary gain outcomes, it is plausible that the no loss has been regarded as the most favorable outcome compared with losses, attributing it a positive (instead of neutral) value (Fig. 5). Hence, it would be important to replicate and extend the current findings in a fully crossed 3 *Emotion* (positive, neutral, negative) × 3 *Monetary outcomes* (gain, no-change, loss) design. Secondly, as we employed erotic images to manipulate positive emotion, we recruited only male participants for this study [see Sescousse et al. (2010) and Buchel et al. (2018) for a similar strategy]. However, for generalizability, it would be essential to replicate the findings in a female cohort using an appropriate set of positive emotional images. Finally, although our RL model analyses indicated some degree of learning associations between actions and outcomes in our choice task, a large variability in learning rates was observed (Fig. 6), suggesting that participants might have engaged different strategies to minimize the monetary losses. Hence, to test the robustness of the current findings, it would be important to examine the interactions between the monetary losses and positive emotion in other task contexts that minimize/control the task strategies being employed.

In conclusion, using a novel experimental design where emotional valence indicated the type of monetary outcome, our study revealed additive effects of positive emotion and monetary loss outcomes in vmPFC, suggesting that the experienced value of monetary loss was not modulated by the valence of the image signaling those outcomes. These findings contribute to our limited understanding of the nature of integrating conflicting outcomes in the healthy human brain with potential clinical relevance in mental disorders such as depression (Schiller et al., 2013; Admon et al., 2015).

## Synthesis

Reviewing Editor: Jibran Khokhar, Western University Department of Anatomy and Cell Biology Decisions are customarily a result of the Reviewing Editor and the peer reviewers coming together and discussing their recommendations until a consensus is reached. When revisions are invited, a fact-based synthesis statement explaining their decision and outlining what is needed to prepare a revision will be listed below. The following reviewer(s) agreed to reveal their identity: Jan Peters.

The reviewer and I have reviewed this manuscript now. Addressing the comments below will help to improve the readability and impact of the manuscript.

In the present fMRI study, the authors probe the integration patterns of monetary loss (vs. no loss) and emotional valence (positive vs. neutral) during feedback processing. The general approach of the study is straightforward, and overall the paper is well-written. The degree to which instructed value (loss vs. no-loss) and intrinsic value (positive vs. neutral) are integrate on the level of reward circuit activity is interesting and novel. I also appreciate the efforts to provide evidence in favour of the null using Bayesian statistics. A weaker point of the paper is the fact that the task was essentially a task with a learning instruction, but no attempts were made to test whether any learning took place.

Major comments

Not sure at what point ROIs were used, and for which (if any) analyses, whole brain correction was performed. Maybe it would help to clarify this in the methods and results section. For example, on page 18 you write that you did not detect any regions with significant (corrected?) interaction effects, but it is not explicitly stated here whether this refers to a whole brain or ROI analysis.

For full transparent reporting, we suggest to add a plot of the subjective ratings that also includes individual-subject data points. Also, this analysis should be done via Bayesian stats to make it comparable to the interaction analysis used to assess evidence in favour of the null for the imaging data.

It's not clear why frequentist statistics were used for the correlation analyses (Table 5), but Bayesian stats for the interaction effects (Table 4).

I think the nature of the task requires additional elaboration and analysis. Based on the methods section, participants were instructed to maximize rewards. Therefore, it seems natural that they would attempt to learn about the associations of actions and outcomes. Notably, there is previous work on the effects of instrinsic stimulus values on reinforcement learning by Chien, Wiehler et al. (2016, J Neurosci) that seems highly relevant here. Thus, it would be important to understand what participants were actually doing / trying to accomplish during the task, and this could be tested by fitting a reinforcement learning model to the choice data. Based on Chien et al., it would be interesting to see whether congruency of stimulus valence and outcome affects the degree of learning / value updating. In its' present form, the paper makes little attempt to understand what processes are actually engaged during task performance, and this would be a straightforward analysis to address this. As fasr as I understand it, there was nothing to be learning on the task, but this is secondary. My point is that participants may have been more (or less) affected by feedback in regulating subsequent decisions, depending on the interaction of stimulus and outcome. This is also supported by the large variance in Chair/Plane choices (Table 7) which suggests that different participants likely enganged very different strategies/policies during the task. A related point is that no attempt is made in the discussion to address the issue of potential learning effects on the task - this needs to be included.

Generally, in my view, the paper needs a limitations section that also addresses issues such as that the design was not fully crossed (i.e. only positive stimuli and loss/no loss outcomes were examined, but not negative stimuli and gain/no gain outcomes), that an all-male sample was tested, etc.

An issue that I was missing in the discussion concerns the different types of in particular aversive stimuli that have been used in this line of work. Aversive / negative cues and events range from losses to aversive stimuli and pain, and the implications of these widely different approaches would need to be addressed in the discussion.

Minor comments

At several points, it should read "less activation" (or better "lower" or "reduced" activation), instead of "lesser activation".
